# Genotyping of *Brucella* strains isolated from humans and cattle of different geographical regions of Pakistan using MLVA‐15

**DOI:** 10.1002/vms3.550

**Published:** 2021-07-10

**Authors:** Raheela Akhtar, Muhammad Muddassir Ali, Asad Ullah, Abdul Muttalib, Kashan Mehboob, Arif Ullah, Nasim Ahmad, Tahir Zahoor Chohan

**Affiliations:** ^1^ Department of Pathology University of Veterinary and Animal Sciences Lahore Pakistan; ^2^ Institute of Biochemistry and Biotechnology University of Veterinary and Animal Sciences Lahore Pakistan; ^3^ Department of Theriogenology University of Veterinary and Animal Sciences Lahore Pakistan; ^4^ Planning and Development Division Pakistan Agriculture Research Council Islamabad Pakistan

**Keywords:** biovars, Brucella, genotyping, MLVA, Pakistan, zoonotic

## Abstract

**Background:**

Prevalence of brucellosis and MLVA genotyping in animals and humans, isolated from different regions of Pakistan was performed. Animals having history of brucellosis from the field and local farms of Bannu, Mardan, Peshawar, Swat, Lahore and Islamabad were selected for blood collection. Humans that work with them were also selected for sampling in this study. Total of 600 samples were taken from cattle and humans and subjected to Rose Bengal plate Test (RBPT) for the initial screening of positive samples. Designed primers of *B.abortus* for cattle and *B.melitensis* for humans were utilised to perform PCR. Culturing and isolation was carried to further to perform MLVA genotyping assay through the selection of two panels of primer markers.

**Results:**

RBPT showed more number of cases of brucellosis in animals and humans compared to the PCR findings. Genotyping findings based upon MLVA‐15 set of markers demonstrated that the isolated strains of *B.abortus* fall in the same clade with strain A1, P8 and A2 from Pakistan and also similar with BCCN#02‐45 strain from India. On the other hand, *B.melitensis* isolated from different districts of Pakistan shared the same clade with BwIM‐AFG 63, BwIM‐AFG 44 strains from Afghanistan and BwIM IRN 37 strain from Iran. Selected VNTR alleles were sequenced for calibration purposes.

**Conclusion:**

It is concluded that *B* *rucella* is prevalent in animals and humans in studied districts of Pakistan. Moreover, A1, P8, BwIM‐AFG 63, BwIM‐AFG 44 and A2 were found the common genotypes in Pakistan.

## Graphical abstract

Biovars; zoonotic; MLVA; Pakistan; *Brucella*; genotyping

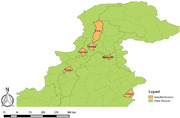

## 1 INTRODUCTION

Brucellosis causes significant economic losses and have zoonotic potential. This disease affects all animals but common and core hosts of *Brucella* pathogen are dogs, camels, goats, sheep, swine, equine, cattle and buffalos. Marine mammals are also affected by brucellosis. Signs and symptoms of *Brucella* disease in Bos taurus are abortion in 3rd trimester of pregnancy and other reproductive problems, in males, it causes infertility and orchitis. It can be transferred from animals to humans through direct contact of infected animals, or by using their by‐products like meat, milk and through infected tissues like uterus, aborted fetus and laboratory exposures (Gwida et al., 2010). In human, this infection can be detected with the symptoms of night sweeting, myalgia, malaise, undulant fever and arthralgia. In incidence of pregnant women, it causes abortion in last trimester plus intra‐uterine fetal loss but human delivery problems are not observed till today (Khan et al., 2001).

Prevalence of brucellosis varies in different regions of Pakistan. Overall prevalence of brucellosis is 21%–26% (Ahmad & Munir, 1995). Seroprevalence was found 5.06% in cattle and 5.49% in buffaloes. Whereas, it was found 1.94% in goats and 1.47% in sheep from Punjab (Nasir et al., 2000). In humans, 16% prevalence have been reported previously from Pakistan (Ali et al., 2018). In our neighbouring country India, overall 16% prevalence has been observed in animals (Deka et al., 2018). Isolation is the typical procedure to diagnose this bacterium but this is time taking procedure and may take weeks. Moreover, isolation procedure requires highly trained personnel and biosafety level‐3. PCR has emerged as the best technique to combat this problem and diagnose the *B* *rucella*. Most commonly representative tissue samples for DNA extraction for the diagnosis of *B* *rucella* can be aborted fetuses, retained placenta and lymphoid tissues (Fekete et al., 1992; Ilhan et al., 2008; Matrone et al., 2009). An innovative technique known as MLVA (Multiple‐Locus Variable number tandem repeat Analysis) is being used in the laboratories to differentiate the strains of *Brucella*. This method is reliable as it can differentiate between those strains of *Brucella* that possess same outlook when performed by any other technique of DNA fingerprinting (Le Flèche et al., 2006, Shevtsova et al., 2019, Vergnaud et al., 2018). Genus *Brucella* has wide variety and various biovars. In earlier studies genus has evidenced to be very monomorphic through the extra than 90% average nucleotide identity among all species (Verger et al., 1985). Due to this similarity, there are difficulties for molecular tests to be able to identify species‐specific individuals competently. Data on genetic diversity on strains of bovine *Brucella* from Pakistan exist (Jamil et al., 2020; Mahmood et al., 2016). One report has been found on the molecular typing of *Brucella abortus* from Pakistan through MLVA‐16 (Ali et al., 2007) but scarcity of data is found regarding strains of human brucellosis. So, this research was performed to find the molecular investigation of human and animal brucellosis from different major districts of Pakistan and to find common *B* *rucella* biovars through MLVA genotyping technique.

## 2 MATERIALS AND METHODS

**FIGURE 1:**
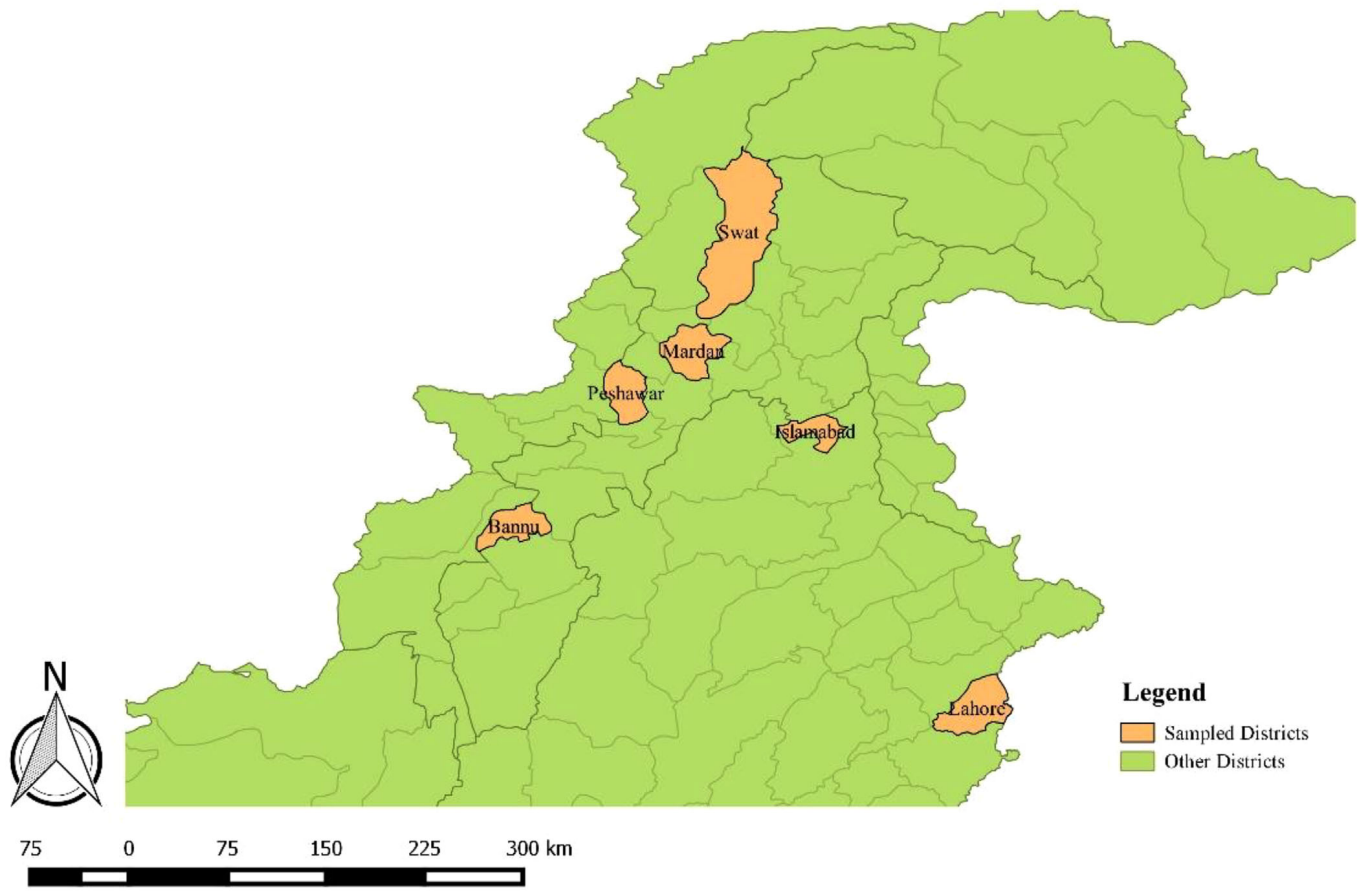
Geographical locations of sampled districts from Pakistan

Random samples (*n* = 300) were collected from cattle with the history of abortion in last trimester, pyometra, metritis, retained placenta, any abnormality in reproductive system or having history related for Brucellosis. Blood samples (*n* = 300) were also taken from those humans who were directly linked with these animals including farmers, workers and milk collector personals. All animals having no history of clinical signs and were apparently healtyhy were not included in sampling. Healthy All Samples were collected February 2017 to March 2019. Major districts of Pakistan, which were investigated, were Bannu, Mardan, Swat, Peshawar, Lahore and Islamabad (Figure 1). These regions were selected based upon the diverse environment and having good population of animals. A total of 6 mL blood was collected from each suspected case, out of which 3 mL blood was placed in EDTA tube and remaining 3 mL was stored in Gel tube for serum collection to perform Rose Bengal plate Test (RBPT). RBPT was performed for Initial screening of brucellosis. Then, RBPT confirmed samples were further processed for PCR confirmation and MLVA genotyping was performed.

### 2.1 Molecular identification

DNA extraction was performed from blood using Gene‐Jet Genomic DNA purification kit (catalogue No. K0722‐250) according to the manufacturer's protocol (Tarique et al., 2017). A Nano Drop of ND‐2000 UV‐V spectrophotometer was used to check the concentration of DNA. The specific sequence regions IS711 for *B. abortus* and *B. melitensis* were targeted for amplification to confirm these organisms having product size 498 bp and 731 bp respectively. Following primers were used, starting from 5', *Brucella melitensis*: forward primer: AAATCGCGTCCTTGCTGGTCTGA; reverse primer: TGCCGATCACTTAAGGGCCTTCAT. Primers for *Brucella abortus*: forward primer: GACGAACGGAATTTTTCCAATCCC; reverse primer: TGCCGATCACTTAAGGGCCTTCAT. Vaccine strain RB51 was used as positive control. And healthy individual free from any disease were taken as negative control. Commercially prepared PCR master mix (Catalogue no. K0171 thermo scientific) was used for amplification. PCR reaction was performed as follows. Initial denaturation was done at 95°C for 10 minutes followed by 35 cycles of denaturation at 95°C for 0.45 seconds, annealing was done at 50°C for 45 seconds, extension was set at 72°C for 45 seconds, and final extension was set at 72°C for 10 minutes (Khosravi et al., 2006). PCR product was than electrophoresed at 100 V for 20 minutes and compared with the DNA ladder (GeneRuler 100 bp – Thermo Scientific). PCR confirmed samples were further cultured on Brain Heart Infusion (BHI) broth to perform MLVA genotyping. Total of 103 samples were cultured. But further, 67 cultures were analysed through MLVA by conceding 5 samples from each city/group.

## 3 MULTI LOCUS VARIABLE NUMBER TANDEM REPEAT ANALYSES (MLVA)

We have used the MLVA assay described by (Le Flèche et al., 2006; Ahmad & Munir, 1995). Briefly, the 15 loci included in the assay consist of two panels for both *B* *rucella* species (*B. abortus* and *B. melitensis*): Panel 1 contained moderately discriminant loci (mini satellites) and panel 2 possessed highly discriminants loci (micro satellites).

PCR confirmed samples were further cultured and DNA was extracted from those cultured samples were used further for PCR amplification with two panels of primers. This amplification was performed in a total volume of 24 μL (12.5 μL master mix, 1.5 μL of each forward and reverse primers, 2 μL *B* *rucella* DNA and 8 μL of water). Amplifications of the samples were done with following conditions. Initial denaturation was done at 96°C for 5 minutes. Thirty cycles of denaturation were performed at 96°C for 30 seconds, primer annealing was done at 60°C for 30 seconds, elongation was at 72°C for 60 seconds and the step of final extension was performed for 5 minutes at 70°C. Gene products were loaded in the wells of 3% agarose gel to analyse tandem repeat. After successful PCR amplification by 15 markers, the product was purified from gel by GeneJET Gel extraction kit (Thermo Scientific) and for Sanger sequencing (1st Base Sequencing Services, Singapore) using Genetic Analyzer 3500xl (Applied Biosystems). Discriminatory power was calculated 0.7345 by Hunter‐Gaston Diversity Index (HGDI). Its value 1 indicated that a typing method is good to differentiate different strain among the population. Zero value indicates that all strains are of identical type in a given population. The value between 0.50 to 1 is good to differentiate different stains in a typing method (Hunter & Gaston, 1988).

### 3.1 Data analysis and sequence submission to NCBI

*Brucella* cases identified through RBPT and confirmed through PCR were expressed in percentages. For comparison VNTR data was obtained from MLVA bank (https://microbesgenotyping.i2bc.paris‐saclay.fr/). JMP v14.0 was used for cluster analysis of VNTRs data. The tree was generated by Ward's minimum variance method. Sequenced data on VNTRs are also submitted to NCBI, which can be retrieved through accession numbers from MW142306–MW142320. Descriptive analysis was performed using R Statistical software PROP.TEST.

## 4 RESULTS

**FIGURE 2:**
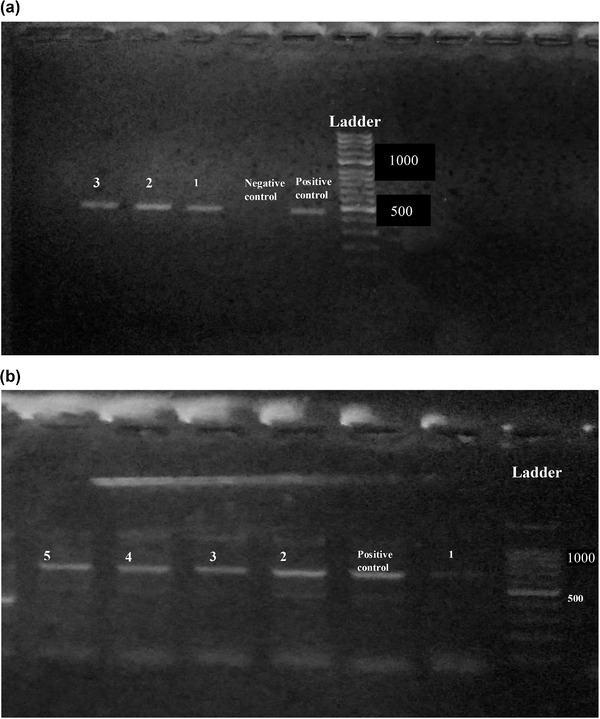
(a) PCR results for *Brucella abortus* having product size of 498 bp. (b) PCR results for *Brucella melitensis* having product size of 731 bp

**FIGURE 3:**
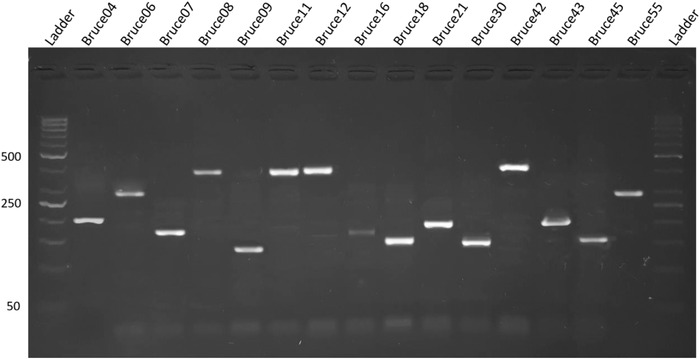
MLVA genomic amplification results of *B. bortus and B. Melitensis* for both panel 1 and 2

**FIGURE 4:**
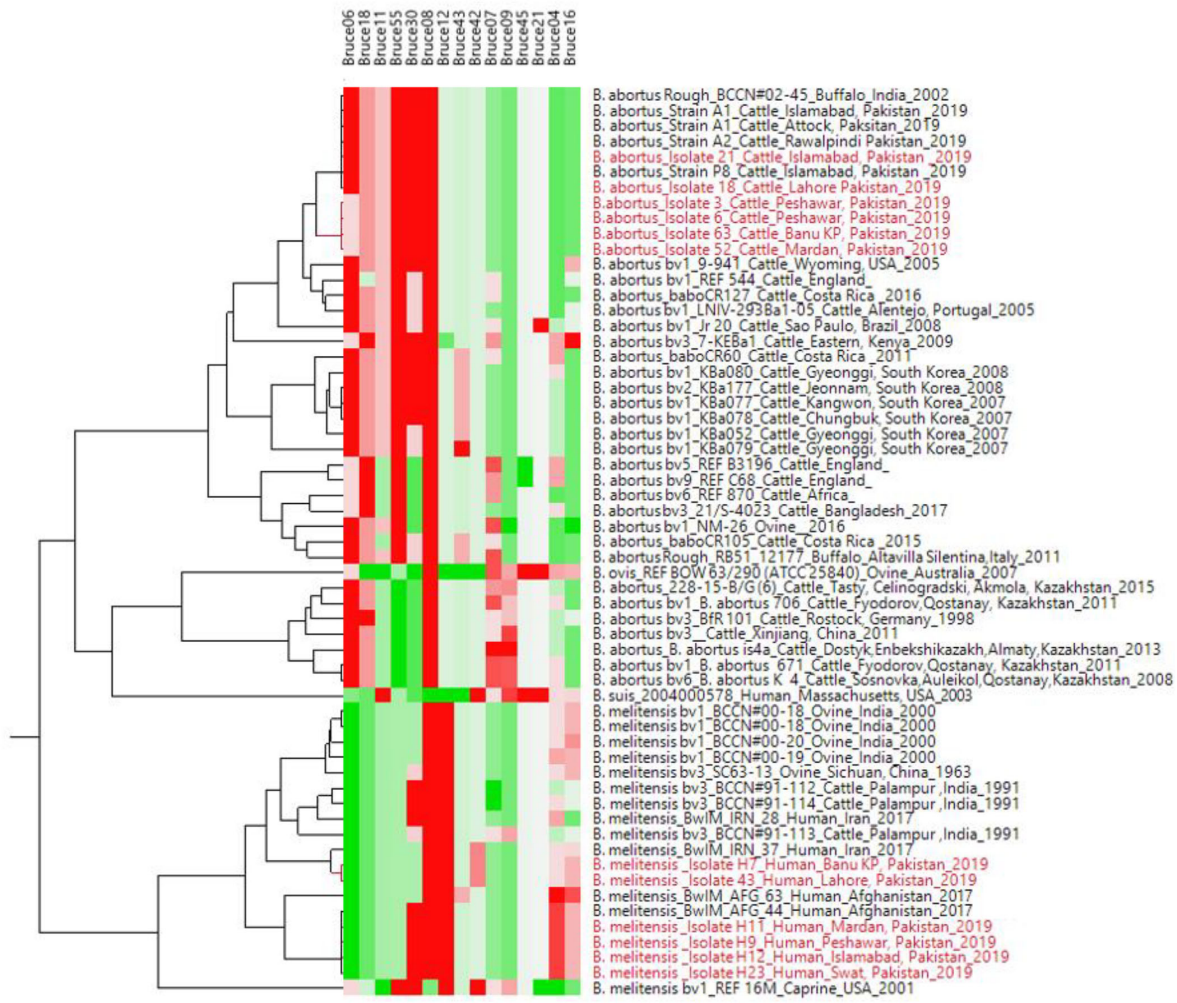
Cluster analysis of VNTR's showing relationships of *B. abortus and B. melitensis* biovars from Pakistan and other *Brucella spp*. isolated from neighbouring countries. The red colour shows highest number of repeats while green shows lowest number of repeats at specific VNTR marker sites

Findings of RBPT, PCR and bacterial isolation are represented in Table 1. Of 600 blood samples, 270 samples were found positive from animals and 122 were found positive from humans in initial screening by RBPT. RBPT showed more numbers of cases of brucellosis in animals and humans compared to the PCR findings. More number of positive cases (76%) were found from Banu district and least number (53%) were found from Islamabad by RBPT test. Confirmation through PCR differentiated the *B. abortus* in cattle and *B. melitensis* in human cases (Figure 2a,b). More number of cases in cattle (*B. abortus*) were found compared to humans (*B. melitensis*). Lots of our positive samples failed for MLVA, and many have partial success. Finally, few of samples successfully amplified for MLVA (Figure 3). Our current 12 isolates were depicting four genotypes. Genotyping findings based upon MLVA‐15 set of markers demonstrated that the isolated strains of *B. abortus* fall in the same clade with strains A1, P8 and A2 from Pakistan and also similar with BCCN#02‐45 strain from India. On the other hand, *B. melitensis* isolated from different regions of Pakistan share the same clade with BwIM‐AFG 63, BwIM‐AFG 44 strain from Afghanistan and BwIM IRN 37 strain from Iran as shown in Figure 4 (supplementary table).

**Table 1:** Total number of cases (%) confirmed through RBPT, PCR and culture for *B. abortus* (cattle) and *B. melitensis* (humans) from different cities of Pakistan

Cities	RBPT		PCR		Culture	
	Positive (*n*) (CI)	Negative (*n*) (CI)	*B. abortus* (*n*) (CI)	*B. melitensis* (*n*) (CI)	*B. abortus* (*n*) (CI)	*B. melitensis* (*n*) (CI)
Banu	76% (65) (0.65–0.84)	24% (20) (0.15–0.34)	67% (20) (0.47–0.82)	33% (10) (0.17–0.52)	67% (16) (0.44–0.83)	33% (8) 0.16–0.55
Mardan	74% (64) (0.63–0.82)	26% (22) (0.17–0.36)	64% (18) (0.48–0.84)	36% (8) (0.15–0.51)	55% (10) (0.31–0.77)	45% (8) (0.22–0.68)
Swat	68% (67) (0.57–0.76)	32% (32) (0.23–0.42)	71% (15) (0.47–0.87)	29% (6) (0.12–0.52)	0% (0) (0.00)	100% (6) (0.51–1.00)
Peshawar	69% (58) (0.54–0.75)	31% (30) (0.24–0.45)	79% (15) (0.53–0.93)	21% (4) (0.06–0.46)	67% (8) (0.35–0.88)	33% (4) (0.11–0.64)
Lahore	57% (70) (0.49–0.68)	43% (48) (0.31–0.50)	74% (14) (0.48 0.89)	26% (5) (0.10–0.51)	75% (15) (0.50–0.90)	25% (5) (0.09–0.49)
Islamabad	53% (68) (0.45–0.63)	47% (56) (0.36–0.54)	81% (13) (0.53–0.95)	19% (3) (0.04–0.46)	77% (10) (0.45–0.93)	23% (3) (0.06–0.54)

*n* = number of sample; CI = confidence interval.

## 5 DISCUSSION

In current study, extensive study on identification of *B* *rucella* biovars in animals and humans were conducted through sophisticated technique. Initial screening by RBPT revealed the prevalence of *B* *rucella* in animals and humans. PCR results showed fewer positive results and further differentiated the *B. abortus* and *B. melitensis* cases. Reason of fewer positive results by PCR could be due to the sensitivity of PCR compared to other conventional diagnostic tests and exclude the false positive results from the samples. We used RBPT as primary test because of larger number of samples, less time consumption and low cost as compared to PCR. During the present study, we have also observed number of untruthful positive results. Its main reason could be antigen purity, storage temperature, contamination and vaccination history of the human and also animals (Doosti & Ghasemi Dehkordi, 2011; Falade, 1978). It could may be because of the existence of few nonspecific agglutinating products of *Brucella*, which had been earlier revealed in the animals carrying no confirmation related to *brucellosis* (Ali et al., 2007).

Seroprevalence studies in Pakistani animals have strong evidence that *Brucella* is prevalent in our country (Ali et al., 2007; Ali et al., 2019; Ali et al., 2018). However, the extensive information on species and biovars of *Brucella* is lacking. In developing countries such as Pakistan, where animals and humans live near to each other, that's why occurrence of *brucellosis* are common in them. Study conducted in Peshawar showed that the seroprevalence of *brucellosis* in Pakistan is 27.65% (Ali et al., 2007). In developing and under developed countries, occurrence of *Brucella* is increasing but in developed countries like Belgium, Malaysia and France are free from *Brucella* disease. In several advanced and developing countries, several plans for screening of *Brucellosis* are followed (Kayombo et al., 2017; Taha et al., 2018). *Brucella abortus* is documented as the main cause of this disease in cattle and buffaloes in bordering countries like Iran and India. In several cases *B. suis* and *B. melitensis* have also been documented in cattle (Sun et al., 2016).

In our study, genotype of *B. abortus* of Pakistan were related to the India and other already reported strains of Pakistan (Ali et al., 2019; Ali et al., 2018). However, *B. melitensis* biovar showed more close resemblance with strains of Iran and Afghanistan. Main reason of this similarity of reported strains of Pakistan with these countries could be due to trade of animals from Pakistan to Afghanistan and Iran or sharing common boundaries. In current study, MLVA‐15 genotyping technique was used for genotype identification through the selection of specific panel of markers. Previously, this technique has already been carried out through the selection of markers. This MLVA technique is employed using combination of justified independent selection of loci which could lead to the phylogenetically relevant and discriminative findings, as earlier observed for many other species (Daugaliyeva et al., 2018; Le Flèche et al., 2006; Shevtsov et al., 2015). MLVA has been performed by taking more than thousands of strains of *B* *rucella* from all parts of the world (Vergnaud et al., 2018) and from central Asia (Shevtsova et al., 2019) to perform its biotyping at larger scale. They revealed unique biovars of *Brucella* and its genotypic expansion among the studied population. (Shevtsova et al., 2019; Vergnaud et al., 2018). Moreover, HGDI was used to discriminate further the genotypes in bacterial population. This technique is widely used as an estimator of genotypes in different bacterial pathogens (Hunter & Gaston, 1988).

The results obtained by the MLVA strengthen the classification of genus *Brucella*. Molecular studies or phenotypic identification can also be found by MLVA. Ease of data exchange is also one of the advantages of MLVA. Data can be summarised, made accessible and queried across the internet by a very simple flat text file containing repeat number of copies for each locus and strain (Daugaliyeva et al., 2018; Liu et al., 2017; Ma et al., 2016; Shevtsov et al., 2015). MLVA typing has another advantage that it's only dependent upon DNA amplicon sizes and lots of electrophoretic techniques are used including manual, agarose gel, low‐cost, high through‐put capillary electrophoresis sequencing machines to interpret the findings (Daugaliyeva et al., 2018; Liu et al., 2017; Ma et al., 2016; Shevtsov et al., 2015). MLVA would become a routine technique or assay for isolation and in the upcoming time, it will be used to generate the international databases containing thousands of MLVA strains. MLVA‐15 assay would be one of these steps in this regard (Ali et al., 2019; Daugaliyeva et al., 2018; Le Flèche et al., 2006; Sayour et al., 2020).

## 6 CONCLUSION

It was concluded that brucella is prevalent in many districts of Pakistan. Humans and animals are equally infected by this bacterium. Common prevailing strains in Pakistan are A1, P8, BwIM‐AFG 63, BwIM‐AFG 44 and A2. Sophisticated diagnostic technique and strict control measures are necessary to eradicate this zoonotic disease.

## CONFLICT OF INTEREST

All author have no conflict of interest.

## ETHICAL PERMISSION

Ethical permission was taken (Dr/1162) from ethical review committee members (Prof. Dr. Muhammad Ashraf and Maryam Sardar) of University and it was according to the institutional guidelines.

## AUTHOR CONTRIBUTIONS

Raheela Akhtar and Nasim Ahmad: conceptualisation, funding acquisition, project administration, and resources. Muhammad Muddassir Ali: supervision, formal analysis, writing original draft, review and editing. Asad Ullah, Abdul Muttalib, and Kashan Mehboob: data curation, investigation, and methodology validation. Arif ullah: data curation, formal analysis, review and editing. Tahir Zahoor Chohan: resources and visualisation.

## Supporting information

Supporting Information

## Data Availability

All data related to the article is presented inside the manuscript.
